# Bruton's Agammaglobulinemia and COVID-19

**DOI:** 10.7759/cureus.11701

**Published:** 2020-11-25

**Authors:** Justin G Hovey, Denise Tolbert, Druhan Howell

**Affiliations:** 1 Internal Medicine/Pediatrics, Acom/Southeast Health, Dothan, USA; 2 Hospital Medicine, Southeast Health Medical Center, Dothan, USA; 3 Allergy/Immunology, Springhill Hospital, Mobile, USA

**Keywords:** agammaglobulinemia, covid-19, convalescent plasma, immunodeficiency, sars-cov-2, ivig

## Abstract

During the SARS-CoV-2 global pandemic, many patients who have co-morbid conditions are considered high risk for morbidity and mortality; however, those who are immunodeficient are at higher risk of becoming seriously ill. In this article, we present a 26-year old male with a history of X-linked agammaglobulinemia who presented to the hospital with fever and chills after exposure to a SARS-CoV-2 positive individual. The patient had a prolonged course in the hospital, but his symptoms improved quickly after receiving convalescent plasma. This case highlights the clinical course of a patient with severe immunoglobulin deficiency and a possible treatment approach for patients with concomitant agammaglobulinemia and COVID-19.

## Introduction

In the United States, X-linked agammaglobulinemia (XLA) is a rare disease with a prevalence of approximately one case per 379,000 live births [1]. XLA is a humoral immunodeficiency described by severe hypogammaglobulinemia usually diagnosed due to recurrent infections in males between 2.6 years of age (with a family history) and 4.5 years of age (without a family history) [1,2]. Individuals with this disease have antibody deficiencies which can lead to recurrent and severe infections [3]. Mutations in Bruton Tyrosine Kinase (BTK) gene found on the long arm of the X-chromosome lead to defective BTK-mediated B-cell signalling and development. Patients affected by this mutation have a paltry number of B-cell lymphocytes in circulation and cannot generate plasma cells; therefore, these patients have decreased production in all classes of immunoglobulin [2-5]. Since XLA patients have a severely decreased humoral response, they have an increased susceptibility to encapsulated organisms and blood-borne viruses such as enteroviruses [2,3]. The lack of humoral response also causes alterations in T-cell response, particularly on immune-memory. These alterations of T-cell memory do not seem to include respiratory viruses like influenza; however, there is little clarity if this extends to SARS-CoV-2 [3,6]. During the SARS-CoV-2 pandemic, case reports of patients with immunodeficiencies are minimal, and clear clinical guidelines for this subset of patients is necessary [7]. Here, we report a 26-year old male with XLA who was hospitalized with COVID-19. The patient had a prolonged hospital course with waxing, and waning symptoms depending on treatments received. Ultimately, the patient did have a good outcome this case aids in our understanding of an appropriate approach to patients with challenging humoral immunodeficiencies and COVID-19.

## Case presentation

A 26-year-old male with a history of X-linked agammaglobulinemia and lower extremity paralysis from previous Guillain-Barré presented to the emergency department (ED) with fever and chills of one-week duration. He denied shortness of breath, chest tightness, cough, or rhinorrhea. His past medical history revealed multiple episodes of community-acquired pneumonia requiring hospitalizations, hypertension, GERD, and Guillain-Barré from an influenza vaccine he received as a child. The patient had been lost to follow-up for nearly two years. Thus he had not received his intravenous immunoglobulin (IVIG) in approximately one year. The patient reported exposure to a SARS-CoV-2 positive individual within his household. The patient's reported medications included metoprolol 25 mg, cetirizine 10 mg, and pantoprazole 40 mg. Upon admission to the ED, the patient's vitals were (a) blood pressure of 132/90, heart rate of 127, oxygen saturation 96% on room air, the temperature of 99.7 degrees Fahrenheit, and respiratory rate of 22. The physical exam revealed a comfortable young male with mild tachycardia and lungs clear to auscultation bilaterally. The patient was stable from a respiratory standpoint. However, his labs revealed elevated transaminases, lactate dehydrogenase, sedimentation rate (ESR), c-reactive protein (CRP), and ferritin. The patient's d-dimer was nearly undetectable (Table 1). COVID-19 was confirmed with a point of care testing.

On admission, the patient received his IVIG after consultation with his primary care physician and a regional allergist/immunologist at a dose of 1g/kg body weight. On post-admission day 2, the patient developed a fever of 102.7 degrees Fahrenheit; therefore, broad-spectrum antibiotics including vancomycin 1g every 8 hours, cefepime 2 g every 8 hours, and doxycycline 100 mg every 12 hours were started as there was a concern for developing post-viral, bacterial pneumonia. Unfortunately, the patient developed a diffuse urticarial rash within 24 hours, so the antibiotics were discontinued. Meropenem 1g every 8 hours was then initiated. The rash reappeared within a few hours, and the antibiotic was discontinued. On post-admission day 3, the patient's fever abated, but he complained of severe dysphagia to solids and pills as well as profuse diarrhoea. He was noted to have oral candidiasis on exam and was started on oral fluconazole 200 mg daily and oral nystatin 6,000 units four times per day. Stool studies, including Clostridium difficile polymerase chain reaction, ova and parasites, and culture were negative. The patient was seen in consultation by gastroenterology for the dysphagia who deferred esophagogastroduodenoscopy secondary to his COVID-19 status. Due to his severe dysphagia, he was empirically started on micafungin 100 mg every day and acyclovir 250 mg every 8 hours per the consultant's request. On post-admission day 4, the patient's fever returned. Levaquin 750 mg daily was subsequently initiated and continued for a total of seven days. A recheck of his labs revealed that the transaminases, ferritin, ESR, CRP, and LDH remained elevated (Table 1). His respiratory status remained unchanged. Likewise, the patient continued to have severe diarrhoea despite treatment with loperamide and probiotics. By post-admission day 11, the patient received 199 ml apheresis convalescent plasma (exact doses unavailable). He remained stable following the infusion. Two days following the plasma infusion, his fever resolved, the ferritin, LDH, CRP, and the transaminases had trended down (Table 1). By post-admission day 14, the patient's diarrhoea improved, and he was discharged home. 

**Table 1:** Laboratory Values LDH- Lactate Dehydrogenase; ESR- Erythrocyte Sedimentation Rate; CRP- C-reactive protein; AST- Aspartate Aminotransferase; ALT- Alanine Aminotransferase; n/a- laboratory values were not checked on those days

Lab (normal values)	Admission	Day 2	Day 4	Day 6	Day 7	Day 9	Day 11	Day 12	Day 14
Ferritin (20-336 ng/dl)	348.1	309.8	1324.3	931.4	997.6	1245.9	774.6	605.8	507.7
D-dimer (0-230 ng/dl)	<200	<200	<200	n/a	200	n/a	<200	n/a	216
LDH (91-200 IU/L)	275	319	427	349	376	361	312	340	249
ESR (0-15 mm/hr)	16	135	111	97	99	95	109	114	121
CRP (0-0.99 mg/dl)	3.49	4.31	2.59	5.57	5.26	5.53	12.86	11.37	5.46
AST (13-39 IU/L)	58	50	201	113	n/a	192	70	56	41
ALT (7-52 IU/L)	56	47	116	90	n/a	118	62	43	33

## Discussion

According to the CDC, the SARS-CoV-2 pandemic has become the third leading cause of death in the United States for 2020. Many of those that survive severe COVID-19 will suffer from life-long morbidity. The most vulnerable patients, in terms of co-morbid conditions, are experiencing the highest rates of morbidity and mortality. Likewise, those with immunodeficiencies are at a higher risk of succumbing to this virus [6]. Case reports on COVID-19 patients with severe immunodeficiencies are needed to inform clear clinical guidelines imperative to patient care [7]. Patients with immunodeficiency face significant challenges during this pandemic. In this case, we were concerned that the patient had not had his IVIG in one year. Knowing his cytotoxic t-cells are still functioning and that he has no way of stimulating an adequate B-cell response, the concern was that we could cause a cytokine storm by infusing IVIG [6]. Also, there is minimal data on morbidity or mortality for those with XLA who are infected with SARS-CoV-2. Therefore, we discussed this case with a regional allergist/immunologist. From that discussion, the decision was made to infuse the IVIG immediately due to the patient's risks of developing overwhelming sepsis from post-viral bacterial infections, and then infuse convalescent plasma once it became available. In our case, this patient seemed to be suffering from diarrhea that we presumed was due to COVID-19 as studies did not reveal any bacterial cause. Following infusion of higher dose IVIG, the patient did not experience any immediate overwhelming immune response. However, he had some worsening pain with swallowing when he was able to generate an immune response to a presumed fungal or herpetic infection, which could be an expected outcome. Fortunately, following the convalescent plasma infusion, the patient's symptoms improved. He defervesced, his diarrhea stopped, and his labs trended down appropriately. Thus, he was able to be discharged home relatively quickly following the infusion and was noted to be at his baseline upon a two-week clinic follow-up.

Given the length of this patient's hospitalization falls in the median range of 4-19 days reported internationally, we have considered that the patient's improvement may have been related to the natural course of the virus [8]. We realize that we cannot definitively state that convalescent plasma changed his clinical outcome. However, his immediate clinical and laboratory improvement following plasma infusion would suggest otherwise. We believe the role convalescent plasma played in this patient's recovery should not be understated. 

## Conclusions

Here, we report a patient with Bruton's who survived COVID-19 with convalescent plasma treatment. Convalescent plasma has been used for over a century; however, our understanding of its use during the SARS-CoV-2 pandemic is incomplete. Despite limited data, studies are ongoing with promising early results. The patient outcome reported cannot be extrapolated to other immunodeficient individuals; however, case studies in patients with rare diseases as reported here may guide clinical approaches. Additional studies using a convalescent serum for COVID-19 are needed. Given the lack of data and the number of immunodeficient patients who are at risk during this pandemic, the use of convalescent serum may be a reasonable approach in those patients with agammaglobulinemia.
