# Correction: Bruton's Agammaglobulinemia and COVID-19

**DOI:** 10.7759/cureus.c43

**Published:** 2021-06-08

**Authors:** Justin G Hovey, Denise Tolbert, Druhan Howell

**Affiliations:** 1 Internal Medicine/Pediatrics, Acom/Southeast Health, Dothan, USA; 2 Hospital Medicine, Southeast Health Medical Center, Dothan, USA; 3 Allergy/Immunology, Springhill Hospital, Mobile, USA

Bruton's Agammaglobulinemia was incorrectly identified as "Burton's Agammaglobulinemia" in the original article title as well as in two locations within the article. These misspellings have been corrected. Cureus and the authors regret the error.

